# Beyond executive functions, creativity skills benefit academic outcomes: Insights from Montessori education

**DOI:** 10.1371/journal.pone.0225319

**Published:** 2019-11-21

**Authors:** Solange Denervaud, Jean-François Knebel, Patric Hagmann, Edouard Gentaz

**Affiliations:** 1 The Center for Affective Sciences (CISA), Campus Biotech, University of Geneva, Geneva, Switzerland; 2 Faculty of Psychology and Educational Sciences (FAPSE), University of Geneva, Geneva, Switzerland; 3 The Laboratory for Investigative Neurophysiology (The LINE), Department of Radiology and Department of Clinical Neurosciences, Lausanne University Hospital and University of Lausanne (CHUV-UNIL), Lausanne, Switzerland; 4 Connectomics Lab, Department of Radiology, Lausanne University Hospital and University of Lausanne (CHUV-UNIL), Lausanne, Switzerland; Utah State University, UNITED STATES

## Abstract

Studies have shown scholastic, creative, and social benefits of Montessori education, benefits that were hypothesized to result from better executive functioning on the part of those so educated. As these previous studies have not reported consistent outcomes supporting this idea, we therefore evaluated scholastic development in a cross-sectional study of kindergarten and elementary school-age students, with an emphasis on the three core executive measures of cognitive flexibility, working memory update, and selective attention (inhibition). Two hundred and one (201) children underwent a complete assessment: half of the participants were from Montessori settings, while the other half were controls from traditional schools. The results confirmed that Montessori participants outperformed peers from traditional schools both in academic outcomes and in creativity skills across age groups and in self-reported well-being at school at kindergarten age. No differences were found in global executive functions, except working memory. Moreover, a multiple mediations model revealed a significant impact of creative skills on academic outcomes influenced by the school experience. These results shed light on the possibly overestimated contribution of executive functions as the main contributor to scholastic success of Montessori students and call for further investigation. Here, we propose that Montessori school-age children benefit instead from a more balanced development stemming from self-directed *creative execution*.

## Introduction

In a professional context where artificial intelligence is expected to surpass humans in the execution of routine tasks, we need to ensure that pedagogical approaches support a workforce capable of *creative executions* to retain its cooperative advantage and benefit from technological advances in autonomy and freedom. Some argue that a human advantage is fundamentally his ability to create; to efficiently execute *individually-driven* thoughts [1]. In the traditional and dominant pedagogical system in Western countries, the metric of school success relies mostly on academic outcomes (e.g., PISA [2]), directly encouraging executive abilities. The drawback of such measures focusing solely on performance and execution is to relegate to the background a more global and integrated child development assessments, which may well also be very important to address the challenges ahead. Conversely, some alternative pedagogical approaches, such as Freinet, Waldorf, Montessori [3, 4, 5], do not target performance per se and tend to address school curricula in a more global and interdisciplinary fashion. They may address academic development differently.

The Montessori pedagogy was born of years of empirical observations of self-directed activities from developing children [6] and feature multi-age classes and a focus on peer-to-peer teaching. Children are free to choose their own learning activities from a specific set of sensory and self-corrective materials, without external feedback such as grades or evaluations [6, 7]. Many pedagogical aspects of the Montessori approach were individually shown to require and train executive functions (EFs), such as goal-directed movement, sequence of gestures to be memorized and repeated in new contexts, and so on [8–10]. Based on preliminary evidence showing that young Montessori schoolchildren (5 years old, on average) achieved higher scores at a card-sorting task [11] than children from traditional schools, it was hypothesized that a Montessori curriculum should more effectively promote EF development [12]. A second longitudinal study of kindergarten children reported some effect on EFs over three years, but not as strong as one could have expected [13]. An exploratory pretest/posttest assessment in a small sample of Montessori preschoolers revealed an improvement that was correlated not with age but with the time spent within the Montessori environment, and beyond the national normed data [14]. However, it cannot be inferred that this advantage is specific to the Montessori setting, as schoolchildren were issued from one single class, this could be a confound with a teacher-effect. While these studies do not report clear and robust effects on EFs, they do not discard this possibility, and investigating EF outcomes in older Montessori students could confirm this hypothesis.

On the other hand, despite no clear differences in EFs, Montessori students were reported to have increased scholastic outcomes, higher creativity skills as well as better well-being at school [11, 13, 15, 16]. Notably, Lillard and Else-Quest (2006) [11] have shown, through a lottery design in U.S. public schools, that children who received a Montessori-based education exhibited cognitive and socio-emotional advantages. These benefits are sometimes debated [17, 18] but seem reproducible as long as the quality and fidelity of pedagogical implementation is observed [19]. In addition, a French study reported advantages for Montessori pupils, regarding both divergent (deriving new elements from a single element) and convergent (integrating diverse elements into a new, single element) creativity over a period of 2 years in children ranging from 6 to 10 years of age [16]. Finally, a more recent and randomized study [13], followed children over the three years of public preschool. Children improved faster in academic achievement, social understanding, and mastery orientation.

In summary, there is core evidence that Montessori schoolchildren score higher on scholastic tasks than traditionally schooled children, but without displaying a definite gain in EFs. Either the EF measures were not yet sensitive enough (measuring combined instead of separated core EFs), or these scholastic performance differences do not rely on EFs alone. In this study, we tested whether reported findings held in another socio-cultural environment, namely Switzerland, while emphasizing the three core EF measures (selective attention, working memory, and cognitive flexibility). We further investigated how creativity, well-being at school, and executive functions mediate academic outcomes. Finally, we assessed the global development of both groups.

We addressed these questions in a large cohort of 201 schoolchildren (*M*_age_ = 9.01 years old, SD = 2.34, 96 girls and 105 boys) through a controlled observational study. As there are no public Montessori schools in Switzerland, and accordingly no option for a lottery design study, we matched pupils from Montessori private schools with peers from traditional public schools controlling for their SES, fluid intelligence, and age.

## Materials and methods

### Participants and procedures

The study’s experimental design was based on existing literature [11, 16]. It was conducted in accordance with the Declaration of Helsinki and ethical committee from the Psychology and Education Faculty, University of Geneva (First approved on the 3rd of December 2015 under the name "Evaluation comportementale des compétences cognitives et émotionnelles chez les enfants de 5–6 ans, 9–10 ans et 12–13 ans scolarisés dans différents environnements pédagogiques -Montessori et Système Traditionnel-"). Teacher participation was voluntary.

Montessori private schools were selected according to the criteria set by the International Montessori Association (S1 Text). For the control group, traditional public schools were selected in specific areas, given the city’s official statistical data on mean salary to include the upper class–salary population only, and were controlled to apply the official local study plan. In total, 21 different classes (13 Montessori classes and 8 traditional classes) from 10 different schools (5 Montessori schools and 5 traditional schools) were included in the study. The 30 teachers who participated were equally experienced (in each group, one teacher was in the early stage of her career, and all others were in the mid-to-late stages of their careers) and trained across systems (all teachers had graduated with an official pedagogical diploma).

Written consent was obtained for each child from his or her parent. Selection criteria included age group (from kindergarten age up to 7 years old, and from elementary age up to 13 years old) and school system (children had to have been enrolled in their school system since the year of their fourth birthday, or for at least 3 years). In total, 208 children were enrolled.

Data from children reported to benefit from psychological support because of learning difficulties (*n* = 2), with low fluid intelligence or low socio-economic status (lower than 2 standard deviations [SDs] from the mean; *n* = 2), outside the target age range (more than 13 years old; *n* = 2) as well as data from nonnative French speakers (as reported by parents or teachers; *n* = 1), were excluded from the study. In total, 201 children from 4.37 to 13.40 years of age (*M*_age_ = 9.01 years old, SD = 2.34, 96 girls and 105 boys) were retained for the study. Ninety-nine (99) participants were schooled in the Montessori educational system (54 girls; Table 1), while 102 were enrolled in the traditional group (42 girls). Descriptive check of age confirmed a bimodal distribution (S1 Fig); children were then assigned to either the kindergarten (*M*_age_ = 5.9, SD = 0.82, 4.4–7.8 years old) or elementary group (*M*_age_ = 10.3, SD = 1.4, 7.6–13.4 years old), according to their current school enrollment (Table 2).

**Table 1:** Study participants.

Control variable (*N* = 201)	Montessori (*n* = 99)	Traditional (*n* = 102)
Age (SD)	8.91 (2.40)	9.10 (2.28)
Age min, max	4.37, 13.37	4.62, 13.28
Gender, # of girls	42	54
Fluid intelligence	30.5 (7.18)	29.4 (6.63)
Socio-economic status	0.70 (0.11)	0.70 (0.12)

**Table 2:** Study participant subgroups.

**Kindergarten**	**Montessori (*n* = 30)**	**Traditional (*n* = 28)**
Age (SD) Min, max	5.93 (0.89) 4.37–7.83	5.87 (0.75) 4.62–7.83
# of girls	16	16
Fluid intelligence	22.8 (8.79)	21.7 (7.26)
Socio-economic status (SD)	0.64 (0.12)	0.70 (0.13)
**Elementary**	**Montessori (*n* = 69)**	**Traditional (*n* = 74)**
Age (SD) Min, max	10.22 (1.53) 7.69–13.4	10.30 (1.21) 7.58–13.3
# of girls	26	38
Fluid intelligence	33.8 (1.98)	32.4 (3.09)
Socio-economic status (SD)	0.73 (0.09)	0.70 (0.11)

Children were tested in schools in a dedicated, separated room. Tasks were either paper or computer based. The total duration of the experiments was of 2 hours, interrupted by brief breaks depending on the participant's fatigue.

### Group comparison

To ensure the homogeneity of the two groups, we controlled for age, gender, socio-economic status, and fluid intelligence.

#### (i) Socio-economic status (SES)

SES was assessed through a parental questionnaire [20] based on education level and both professional situation and category that 79% of parents filled out.

#### (ii) Fluid intelligence (FI)

FI evaluation was made with the help of a black-and-white version of Raven's Progressive Matrices (PM-47) test [21] (S2 Fig). The task comprised 36 items. For each item, an incomplete matrix was presented, and the child was asked to identify the missing element that completes the matrix. Each correct item granted 1 point, with the maximum score being 36.

### Scholastic assessment

Each child's global scholastic development was evaluated using well-established metrics based on four aspects (i–iv): executive functions, academic outcomes, well-being at school, and creativity.

#### (i) Exectuive Functions (EFs)

EFs were evaluated with the help of two different types of tasks: (a) selective attention (inhibition) and (b) cognitive flexibility measures were derived from reaction time (RT) of the Flanker fish task (a child-friendly version of the flanker task, where arrows are replaced by fishes) [22]. In this particular experiment (performed using Presentation^®^ software), the child was asked to indicate the orientation of fish (replacing the pointing arrow) by pressing keys during three different blocks. Rules were switched from the first block (focus on the fish at the center of a line of five blue fishes—17 trials) to the second block (focus on the four fish flanking the central one, all pink—17 trials). The final block randomly mixed both instructions (line of five blue fish or five pink fish for 45 trials). Response time limit was 2,000 ms for children up to 6 years old and 1,500 ms for older children. Trials with valid RT (within 2 SD) were computed as follows: for selective attention (inhibition), mean RT of congruent trials were subtracted from mean RT of incongruent trials within the first block. For cognitive flexibility, switching was computed as the mean of RT differences between successive blocks with a switch in the rules (i.e., from a line of blue fish, to a line of pink fish, last block only). (c) Working memory update was measured from the Ascending Digit (up to 6 years old) or Digit-Letter (more than 6 years old) span tasks (item from the WISC-IV)[23]; the child was asked to listen to and memorize a string of mixed digits or mixed digit-letters, and to repeat them in an orderly ascending manner. The game started with a two-digit string; when the child successfully performed two trials in a row, an extra digit was added to the string. If the child missed a trial, a digit was removed from the string. If the child missed either three trials in a row or three trials at a single level, the game ended. The final score was age standardized.

#### (ii) Academic outcomes

Academic outcomes were assessed using both literacy and numeracy standardized tasks. Younger children (up to 6 years old) were evaluated through oral comprehension [24], early reading competence [25], and verbal problems [26]. (a) Oral comprehension: 27 items from Pierre Lecocq’s “Epreuve de Compréhension Syntaxico-sémantique” (E.CO.S.SE) to evaluate oral comprehension were selected. Children were told a sentence and had to select among four pictures the one corresponding to that sentence. Correct responses were summed to obtain the final score (maximum 25; S3 Fig). (b) Early reading competence: First, phonemic and syllabic awareness was measured using items as cited in Gentaz et al. (2013). The child was told a pseudoword and had to repeat the same pseudoword without the first syllable (10 items) or the first phoneme (24 items). Length and difficulty increased throughout the task (maximum score, 34). Second, each child performed a decoding task (Word attack); reading 30 pseudowords within 1 minute (maximum score, 30). Accuracy across language tasks was summed and expressed as a percentage. (c) Verbal problem: children were told orally 10 different verbal problems and had to report their answer each time (S4 Fig). Accuracy (0 or 1) was computed, the final score being the sum with a maximum of 10 and expressed as a percentage of accuracy.

For older children, we evaluated language and mathematical skills through standardized competence scales [27]. (a) Language competence: Based on a story the child was asked to first read, several skills were successively tested: reading comprehension (questions on the story), grammar, and spelling tests. The maximum score was 100% of correct answers. (b) Mathematical competence: The child had to perform some arithmetical, logical, and geometric tasks. The maximum score was 100% of correct answers.

#### (iii) Self-reported well-being at school

Well-being at school was evaluated through questionnaires. Children up to 6 years old answered the “Feeling about School” questionnaire [11] using a graduated faces scale (from a very sad face to a very happy one) corresponding to a 5-level Likert scale. Older children filled out the Buss and Plomin questionnaire for the sociability measure [28]. Children answered statements about their feelings using a Likert scale ranging from 0 to 4. The final score was expressed as a percentage.

#### (iv) Creativity

Creativity was measured using both divergent and convergent abstract drawing items from a standardized test [29]. (a) Divergent creativity: The child was asked to draw as many different drawings as possible from one imposed abstract form, within a time frame of 10 minutes. The final score was the sum of all valid creations, where the initial imposed abstract form was correctly integrated within a new concept. (b) Convergent creativity: The child was asked to pick at least three different abstract forms out of eight and to create one new drawing that combined them, within a time frame of 15 minutes. Drawings were blindly scored by three different judges following the referenced scale (maximum of 7, from 1 = very poor creativity to 7 = highly creative). Criteria were originality and storytelling of the drawing. The final score was expressed as a percentage.

### Statistical analysis

Statistical analyses were computed using R, and, in part, jamovi (Version 0.9) Computer Software.

#### Group comparison

Prior to group comparison, statistical *t* tests were run on the control variables (age, fluid intelligence, and socio-economic status) to ensure group homogeneity (S1 Table).

#### Scholastic outcomes

**T tests.** Assuming a selection bias, scores per task were tested statistically using bootstrapping Yuen *t* test [30] with 20% trimming and 600 repetitions for bootstrapping. This test was used to determine significant differences between the two groups of schoolchildren (Montessori vs. traditional) at the two age levels (kindergarten and elementary), with a false discovery rate (FDR) *p*-value correction at *q* = 0.05. Additionally, we controlled for age by running ANCOVA on each measure with age as a covariate.

**Multiple mediation model.** *Z*-scored data from the same cognitive measure (executive functions, academic outcomes, well-being at school, or creativity skills) were averaged across subjects. A multiple mediator model was built and computed on the pooled data to evaluate the effects of multiple factors (executive functions, creativity skills, well-being at school) simultaneously on academic outcomes, when the predictor was school system (contrast Montessori-Traditional). The full model was *Academic outcomes ~ executive functions + well-being at school + creativity skills + system*, and the mediator model was *executive functions~system (M-T)*, *well-being at school~system (M-T)*, and *creativity skills~system (M-T)*. We used the large sample *z*-test of the mediated effect, known to be slightly more accurate than the Sobel test, with 1,000 bootstrap repetitions (percentile method) [31].

**Radial plot.** Finally, a radial plot was designed to qualitatively represent the scholastic development of each child and the mean for both groups (Montessori or traditional) with the pooled dataset. There were four axes in the radial plot; each edge standing for the maximal score possible for the core skills (academic outcomes, EFs, creativity, and well-being), and the center standing for the minimal score for all the skills. Each child’s averaged *z*-score was reported as a distance along each axis and joined between axes.

## Results

Children were proficient at all tasks, and no one was excluded due to missing data or outlier outcomes. The scores were individually computed and reported before the statistical comparison between the two groups at both school-level, controlling for age. We then built the multiple mediation model to investigate the relationships between EFs, creativity, well-being at school, and academic outcomes. Finally, we plotted a qualitative measure of global scholastic development through the radial representation.

### Scholastic outcomes

At kindergarten age, between group comparison revealed that, even when controlling for age, Montessori schoolchildren score higher than same-age children from traditional schools on language, math, well-being, working memory, convergent and divergent creativity tasks (Table 3, top panel). At elementary age, results revealed that language, math, working memory, convergent and divergent creativity scores were higher in the Montessori schoolchildren than in same-age children from traditional schools, even when controlling for age (Table 3, bottom panel). Our findings are of medium to large effect sizes (Cohen’s *d*), that are at least comparable to previous studies comparing Montessori and traditional schoolchildren (Table 3, right column).

**Table 3:** Scores per age level and group (mean, SD), and statistics.

**Kindergarten**	**Montessori mean (SD)**	**Traditional mean (SD)**	**Yuen’s test bootstrapped (*p*-values FDR corrected)**	**Main effect of pedagogy when controlling for age (ANCOVA)**	**Effect size** **Cohen’s *d***	**Effect size Cohen’s *d* from randomized studies [11, 13]**
**Language (%)**	**66.1 (26.7)**	**51.8 (23.8)**	**2.05, p = 0.06**	**5.26, p = 0.026**	0.56	0.44 (Letter-Word) & 0.63 (Word Attack); 0.36 & 0.41 (Academic achievement at time 1 and time 2)
**Math (%)**	**45.1 (27.8)**	**23.9 (31.0)**	**3.52, *p* = 0.012**	**8.66, *p* = 0.005**	**0.72**	0.55 (Applied problem)
**Well-being at school (%)**	**87.2(12.0)**	**75.8 (13.9)**	**3.69, *p* = 0.008**	**11.13, *p* = 0.002**	**0.88**	
**Convergent creativity (score)**	**3.88 (1.49)**	**2.74 (1.27)**	**3.54, *p* = 0.013**	**11.8, *p* = 0.001**	**0.82**	
**Divergent creativity (score)**	**6.63 (4.32)**	**3.36 (2.72)**	**2.89, *p* = 0.016**	**13.6, *p* < 0.001**	**0.90**	
**Working memory (score)**	**5.30 (1.85)**	**4.16 (1.56)**	**3.13, *p* = 0.016**	**7.01, *p* = 0.010**	**0.66**	0.61 (Dimensional Card Sort); 0.35 (at time 3 for the Head-Toes-Knees-Shoulders and Copy-Design tasks)
Selective attention (ms)	74.5 (203)	144 (245)	–1.77, *p* = 0.100	1.29, *p* = 0.260	0.31	
Cognitive flexibility (ms)	46.7 (104)	31.0 (76.7)	0.22, *p* = 0.380	0.355, *p* = 0.554	0.17	
**Elementary**	**Montessori mean (SD)**	**Traditional mean (SD)**	**Yuen’s test bootstrapped (*p*-values FDR corrected)**	**Main effect of pedagogy when controlling for age (ANCOVA)**	**Effect size** **Cohen’s *d***	**Effect size Cohen’s *d* from randomized studies**
**Language (%)**	**74.4 (14.8)**	**57.6 (26.7)**	**3.74, *p* = 0.004**	**29.0, *p* < 0.001**	**0.78**	
**Math (%)**	**66.1 (25.0)**	**45.1 (26.7)**	**4.28, *p =* 0.003**	**30.6, *p* < 0.001**	**0.81**	
Well-being at school (%)	65.7(20.7)	63.4 (19.7)	0.42, *p* = 0.77	0.60, *p* = 0.442	0.12	0.54 (positive school feeling)
**Convergent creativity (score)**	**5.13 (1.46)**	**3.53 (1.51)**	**6.07, *p =* 0.003**	**43.93, *p* < 0.001**	**1.07**	0.71 (Creativity of narrative)
**Divergent creativity (score)**	**10.8 (4.08)**	**7.42 (4.65)**	**4.61, *p =* 0.003**	**22.76, *p* < 0.001**	**0.76**	
**Working memory (score)**	**7.32 (2.12)**	**6.29 (2.35)**	**1.99, p = 0.053**	**7.79, p = 0.006**	0.46	
Selective attention (ms)	21.8 (85.4)	7.18 (87.6)	1.38, *p* = 0.24	0.94, *p* = 0.335	0.17	
Cognitive flexibility (ms)	51.3 (68.6)	43.7 (54.5)	0.12, *p* = 0.900	0.44, *p* = 0.508	0.12	

### Multiple mediation model

**Fig 1:**
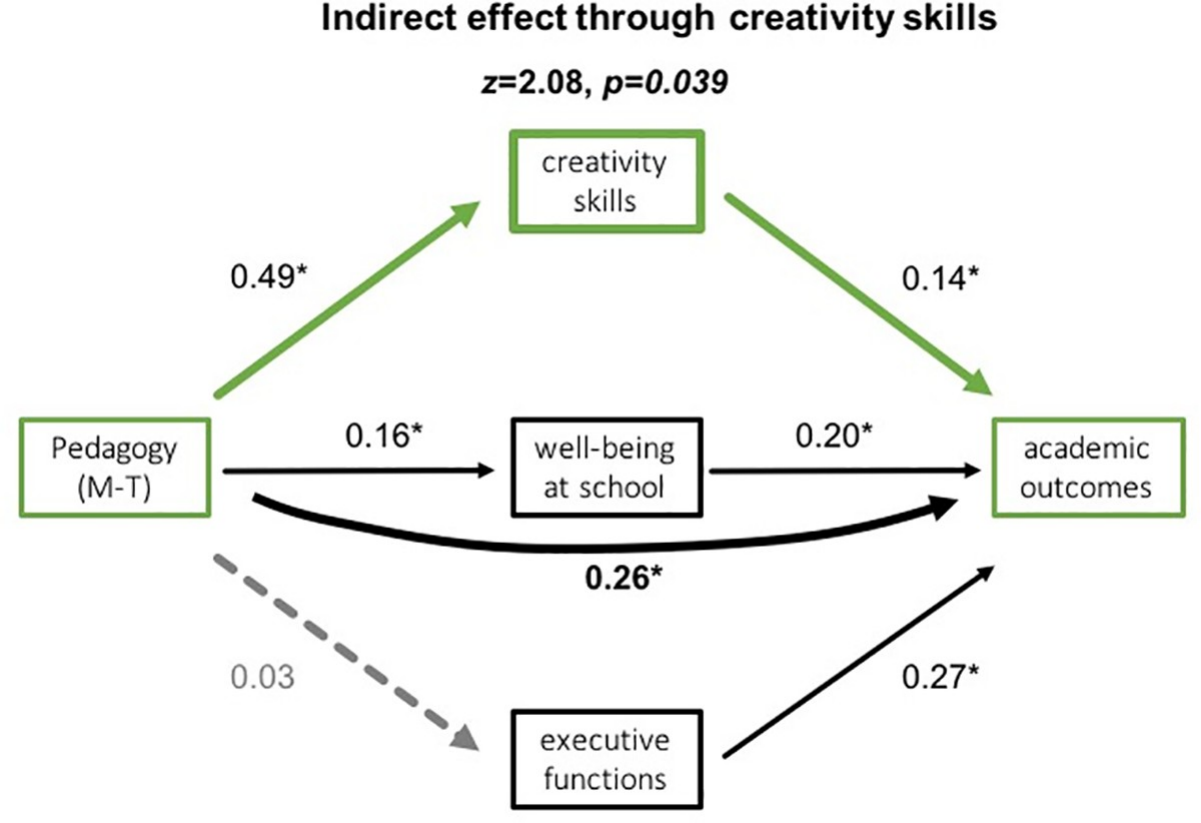
Multiple mediation model (according to [31]) for the indirect effect of children’s school system (Montessori vs. traditional) via multiple mediators (executive functions, well-being at school, and creativity skills) on academic outcomes. The only significant (*z* > 2) indirect mediation effect on academic outcomes was creativity skills in Montessori schoolchildren (green path). The standardized solution coefficients (ß) and significant *p*-values < 0.05 (depicted with a star) are reported next to related path.

There was a significant indirect effect of creativity skills only on academic outcomes, *z* > 2, *p* = 0.04. As Fig 1 illustrates, for Montessori schoolchildren, the standardized regression coefficient between system and creativity as well as creativity and academic outcomes were statistically significant (*p* < 0.001 and *p* = 0.036, respectively). The standardized indirect effect was ß = 0.39 (*p* < 0.001), with partial mediation of Montessori system by creativity on the academic outcomes. Of note, the standardized regression coefficient between system and executive functions was not significant (*p* = 0.18) (S2 Table).

### Radial representation

**Fig 2:**
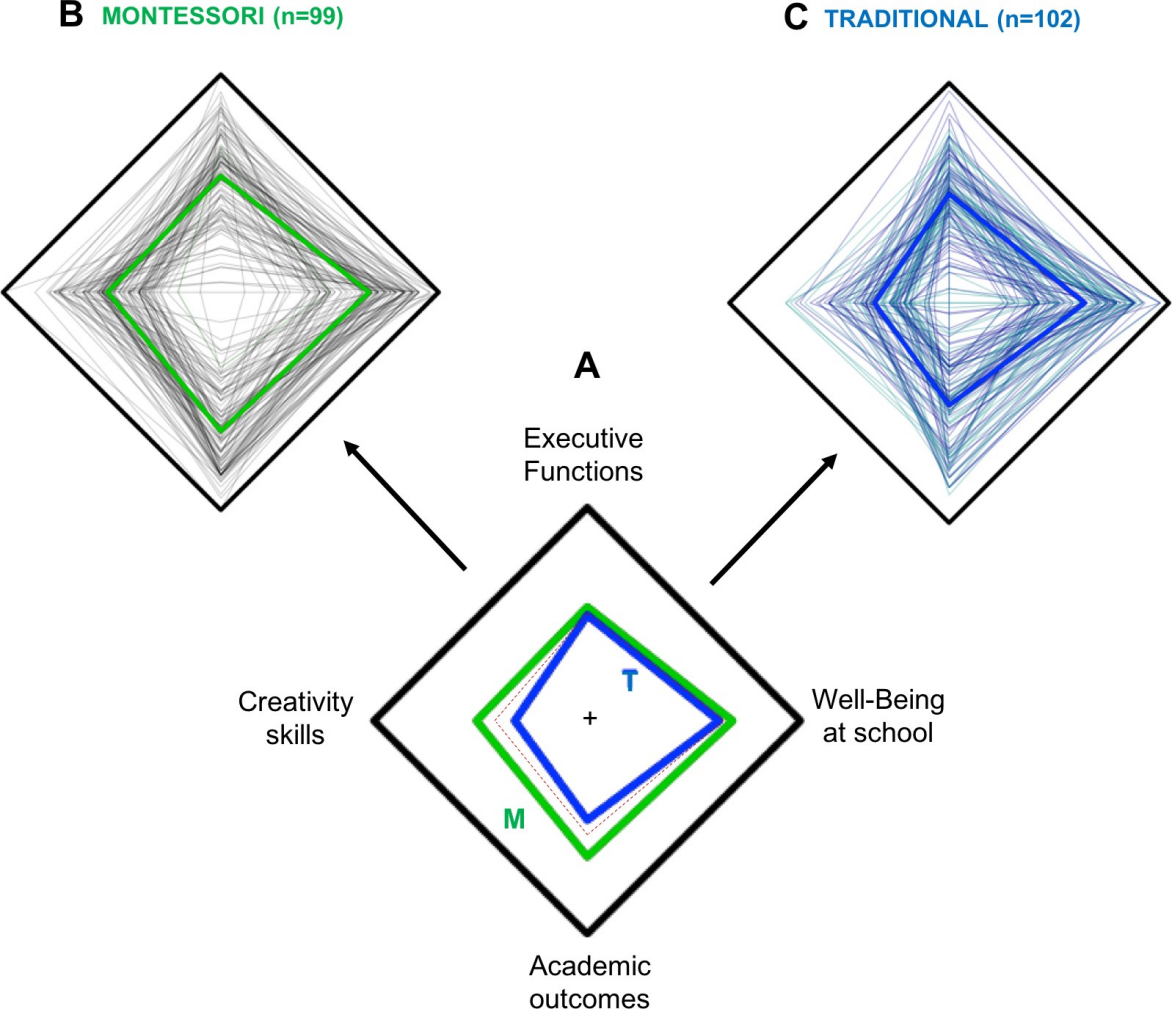
Radial qualitative representation of the four different cognitive measures: executive functions, creativity skills, well-being at school, and academic outcomes, each located at a summit. The scales depend on the measured cognitive skill; however, all run from the minimum at the center to the maximum at the border of the square. Individual results are represented with a thin line (Montessori schoolchildren on top left 2B, and control on the top right 2C), and mean for each group is reported with a bold line in the central square 2A, where the dotted red line marks the 0 of each cognitive measure’s z-score scale. Montessori (M) depicted in green, traditional (T) in blue. Group differences (M vs. T) are observed for creativity skills and academic outcomes.

Fig 2A depicts the radial representation of the group means. Each core skill *z*-score center is tracked with the red line, allowing a visual assessment of mean global development. The pattern shows that only creativity skills and academic outcomes differ between groups in favor of Montessori schoolchildren. Fig 2B (Montessori schoolchildren) and Fig 2C (traditional schoolchildren) are radial plots, where individual outcomes are depicted; of note, there is a visible difference on the “creativity” corner.

## Discussion

We evaluated cognitive measures that were studied separately in previous works done in the field, comparing Montessori with traditional schoolchildren on scholastic, creativity and well-being outcomes [11, 13, 15, 16].

Regarding scholastic and creativity scores, our findings corroborate previous studies [11, 16] but in a different cultural environment, suggesting that some of the measured effects could reflect the child pedagogical experience in a Montessori setting. Kindergarten Montessori schoolchildren also reported a better sense of well-being at school than schoolchildren from traditional pedagogy. This is in line with previous studies using the same tasks; however, we did not find a similar difference among elementary schoolchildren. Based on the existing literature reporting that the Montessori pedagogy promote students’ sense of belonging to the school [11, 15], with higher autonomy usually leading to well-being [32], this result is contrary to our expectations. This may reflect more generally a developmental shift in how schoolchildren orient and evaluate their social interest at school; from the teacher at kindergarten-age to their peers from 6 years old onwards. The general attenuation in well-being with age may thus reflect the usual appearance of socio-cognitive conflicts in children and/or the social bias of self-reported questionnaires [33].

Concerning EFs, which include cognitive flexibility, working memory update, and selective attention (inhibition) according to Miyake's model [34], no difference was found between school settings. The exception was for working memory, which was found to be different in favor of Montessori students. As opposed to cognitive flexibility and inhibition, which were measured based on RT (speeded response task) through a computerized task, working memory was measured as a score (no time restriction). Time limit and/or the screen interface could artifact the outcomes, since Montessori schoolchildren are not accustomed to this type of activity within their school environment, nor to work under time pressure. Previous studies making use of screen-free tasks with no account for RT reported advantages for the Montessori schoolchildren. For example, the Head-Toes-Knees-Shoulders and Copy-Design tasks showed an improvement over two years in Montessori schoolchildren compared to controls from traditional schools [13]. Either these tasks pull more on the child’s working memory capacity, or time/computer constrains their actual competences. We further addressed the issue of timing by looking at the error-rate of the flanker task instead of RT, but none of these analyses revealed group differences (p>0.5). Another possible explanation for the absence of a clear global EF difference in our cohort could also stem from the known relationship between EFs and SES [35]. Indeed, in the context of our study, participants come from relatively high-income family environments, which likely influenced their EF capacity in a similar way. In addition, these children attend schools with high-quality settings, where great emphasis was placed on EF trainings in the last decade.

While there are no differences in EFs, their self-monitoring could still differ. Empirical studies describe Montessori schoolchildren with the capacity for a deep concentration state [9,18], which certainly rely on combined self-regulatory features rather than just selective attention capacity (high focus). In this context, it would be of interest to measure self-directed EF [36], as the Montessori children are trained for more autonomous thinking behaviors that could promote more “intrinsically” driven executive control, and also explain their higher creativity skills.

This was further explored through the multiple mediator analysis. Our data shows that beyond EFs, creative competencies specifically modulate academic success in Montessori schoolchildren, suggesting good execution of self-generated ideas. This aspect of EFs is currently understudied in the framework of academic outcomes in school years.

Finally, pupils attending a Montessori school were shown to have a more balanced global development. This may play a key role in promoting academic performance. This finding raises the question of the limit of emphasizing a unique aspect of scholastic development, such as EFs. In fact, cognition with less control (lower EFs) as during the childhood years presents many advantages, such as faster learning rate and higher creative abilities [37]. Seeking for cognitive performance may be at the cost of qualitative and long-term learning[38]. Accordingly, expecting schoolchildren to maintain a high level of selective attention, or placing too much emphasis on other core EFs, may well be counterproductive and impair the individual’s innate capacity for learning and creative execution abilities.

More broadly, one can wonder whether educating and directing competencies in isolation does not prevent schoolchildren from making connections or unrelated links later on, and thus prevent from nurturing individual creative thinking. Indeed, creativity is frequently attributed to genius or pure talent—an innate spark found only in the Albert Einsteins, Pablo Picassos, or Steve Jobses of this world. However, creative thinking is a fundamental competency, present in all of us to different degrees, and something that can be nurtured. We need to address and educate *creative execution abilities*, not simply by allocating more hours for painting or crafting within curricula (there is little of these activities within the Montessori education, for instance), but rather by investigating which aspects of pedagogical approaches fostering creativity, such as Montessori, make it possible for schoolchildren to grow this way of thinking. We suspect that it results from a combination of features more than one; such as using more naturalistic activities that are inherently inter-disciplinary, interacting with peers from different ages, making a choice amongst different activities, taking the lead over projects, or seeking for answers and solutions on their own. These pedagogical aspects are not easy to capture scientifically and will highly benefit from extensive multimodal research in the future.

The main limitation of our study is the fact that, due to local policies in Switzerland, the Montessori classes included in the study are all in private schools, whereas the traditional schools are public. We chose public schools in areas of similar wealth to that of Montessori school candidates and controlled for their SES. This may constitute, despite all precautions, a selection bias. Nevertheless, our basic findings are in agreements with the two existing randomized studies made in public Montessori schools [11, 13], suggesting that this bias may be weak or negligible and that the observed effect is mainly attributable to schooling differences. However, in our effort to match the schoolchildren based on their SES, we did not account for the possible bias that parents enrolling their children within Montessori curricula could themselves present higher creative thinking. If so, interactions with their child could also influence the higher level of creativity measured in our study. Further studies should be conducted to deepen these findings and would benefit either from a longitudinal or a lottery design study instead of the use of matched controls to clarify some of the uncertainty raised in this discussion.

## Supporting information

S1 Text School selection criteria. (PDF)

S1 Table Control variables’ t-Test. Age, Fluid Intelligence (FI) and socio-economic status (SES).
(PDF)

S2 Table Mediation model. (PDF)

S1 Fig Age distribution. (PDF)

S2 Fig Fluid Intelligence was measured with the help of the Raven matrices task, examples displayed here. (PDF)

S3 Fig Oral comprehension, examples from the E.C.O.S.S.E task. (PDF)

S4 Fig Verbal problem examples. (PDF)
