# Supplementary material for: Beyond executive functions, creativity skills benefit academic outcomes: Insights from Montessori education
Source: PLoS One. 2019 Nov 21;14(11):e0225319. doi: 10.1371/journal.pone.0225319 (PMC6874078; doi:10.1371/journal.pone.0225319)
Supplement: S1 Text — (PDF) [file pone.0225319.s001.pdf]

The criteria set by the International Montessori Association (AMI) (https://montessori-ami.org):

(i) all teachers were AMI trained,

(ii) a complete set of Montessori material was available in each classroom,

(iii) children had a 3-hour continuous working time, and

(iv) there were at least 3 different age-levels per class.

The official local study plan for traditional public schools strictly implies, as from 6 years old:

(i) frontal teaching,

(ii) tests and formal evaluations,

(iii) breaks every hour, and

(iv) one age-level per class.

N.B. In Switzerland, it is quite common to have two part-time teachers that share the lead of one class, which was the case for many of the public traditional classes included in the study. In addition, Montessori classes had often two teachers per class, if the latter was large (according to the Swiss law).
