# Supplementary material for: Beyond executive functions, creativity skills benefit academic outcomes: Insights from Montessori education
Source: PLoS One. 2019 Nov 21;14(11):e0225319. doi: 10.1371/journal.pone.0225319 (PMC6874078; doi:10.1371/journal.pone.0225319)
Supplement: S1 Table — Age, Fluid Intelligence (FI) and socio-economic status (SES). (PDF) [file pone.0225319.s002.pdf]

**Control variable**	**t**	**df**	**p**	**Cohen’s d**
Age	-0.59	199	0.56	-0.08
FI	1.09	199	0.28	0.15
SES	0.92	156	0.36	0.15
