# Supplementary material for: Beyond executive functions, creativity skills benefit academic outcomes: Insights from Montessori education
Source: PLoS One. 2019 Nov 21;14(11):e0225319. doi: 10.1371/journal.pone.0225319 (PMC6874078; doi:10.1371/journal.pone.0225319)
Supplement: S2 Table — (PDF) [file pone.0225319.s003.pdf]

Models Info

Mediators Models		
	m1	Creativity skills ~ Pedagogy
	m2	Well-being at school ~ Pedagogy
	m3	Executive functions ~ Pedagogy
Full Model		
	m4	Academic outcomes ~ Creativity skills + Well-being at school + Executive functions + Pedagogy
Indirect Effects		
	IE 1	Pedagogy ⇒ Creativity skills ⇒ Academic outcomes
	IE 2	Pedagogy ⇒ Well-being at school ⇒ Academic outcomes
	IE 3	Pedagogy ⇒ Executive functions ⇒ Academic outcomes

Indirect and Total Effects

				95% C.I. (a)				
**Type**	**Effect**	**Estimate**	**SE**	**Lower**	**Upper**	**β**	**z**	**p**
Indirect	**Pedagogy1 ⇒ Creativity skills ⇒ Academic outcomes**	**0.0304**	**0.01465**	**-0.00257**	**0.0562**	**0.0684**	**2.07**	**0.038**
	Pedagogy1 ⇒ Well-being at school ⇒ Academic outcomes	0.0141	0.00793	0.00126	0.0324	0.0317	1.78	0.076
	Pedagogy1 ⇒ Executive functions ⇒ Academic outcomes	0.0115	0.00970	-0.00644	0.0324	0.0258	1.18	0.238
Component	**Pedagogy1 ⇒ Creativity skills**	**0.1796**	**0.02208**	**0.13393**	**0.2228**	**0.4870**	**8.13**	**< .001**
	**Creativity skills ⇒ Academic outcomes**	**0.1691**	**0.08028**	**-0.01448**	**0.3086**	**0.1405**	**2.11**	**0.035**
	**Pedagogy1 ⇒ Well-being at school**	**0.0630**	**0.02812**	**0.00627**	**0.1187**	**0.1585**	**2.24**	**0.025**
	**Well-being at school ⇒ Academic outcomes**	**0.2235**	**0.06837**	**0.08783**	**0.3556**	**0.1999**	**3.27**	**0.001**
	Pedagogy1 ⇒ Executive functions	0.0385	0.02980	-0.02243	0.0934	0.0949	1.29	0.196
	**Executive functions ⇒ Academic outcomes**	**0.2974**	**0.06360**	**0.17958**	**0.4324**	**0.2719**	**4.68**	**< .001**
Direct	**Pedagogy1 ⇒ Academic outcomes**	**0.1167**	**0.02993**	**0.06126**	**0.1801**	**0.2629**	**3.90**	**< .001**
Total	Pedagogy1 ⇒ Academic outcomes	0.1726	0.02905	0.11563	0.2295	0.3873	5.94	< .001

Note. (a) Confidence intervals computed with method: Bootstrap percentiles Pedagogy 1 stands for M-T.
