# Supplementary figures and images for: Beyond executive functions, creativity skills benefit academic outcomes: Insights from Montessori education
Source: PLoS One. 2019 Nov 21;14(11):e0225319. doi: 10.1371/journal.pone.0225319 (PMC6874078; doi:10.1371/journal.pone.0225319)

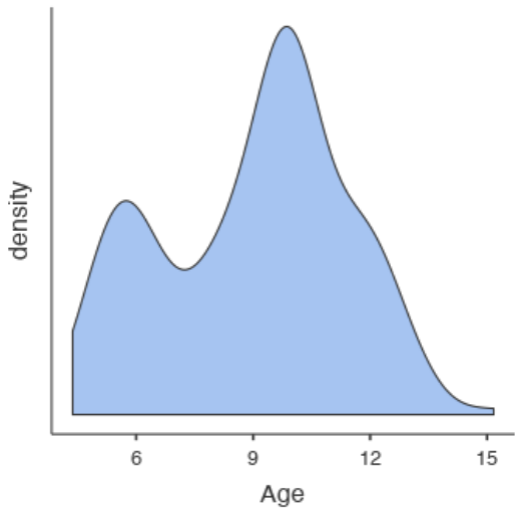

Supplement: S1 Fig — (PDF) [file pone.0225319.s004.pdf]

A5

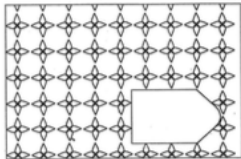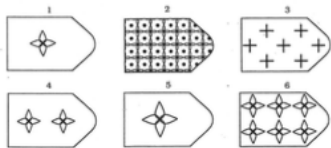

A6

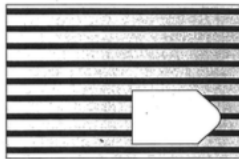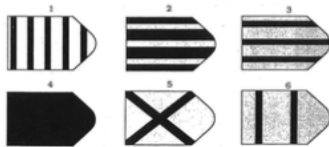

A7

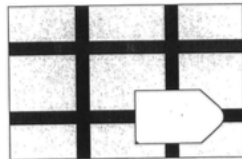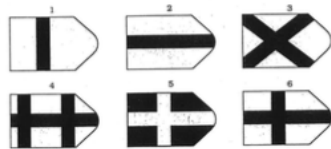

Supplement: S2 Fig — (PDF) [file pone.0225319.s005.pdf]
