# Supplementary material for: Beyond executive functions, creativity skills benefit academic outcomes: Insights from Montessori education
Source: PLoS One. 2019 Nov 21;14(11):e0225319. doi: 10.1371/journal.pone.0225319 (PMC6874078; doi:10.1371/journal.pone.0225319)
Supplement: S3 Fig — (PDF) [file pone.0225319.s006.pdf]

E.CO.S.SE	Examples of sentence for oral comprehension (CO)	Pictures
**Active sentences**	**OC**- La fille pousse le cheval.<br>*The girl pushes the horse*	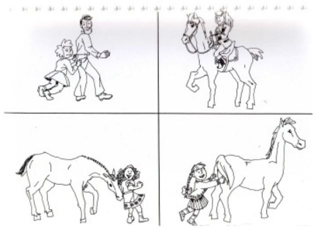
**Passive sentences**	**OC** - La fille est poursuivie par le cheval.<br>*The girls is pursued by the horse*	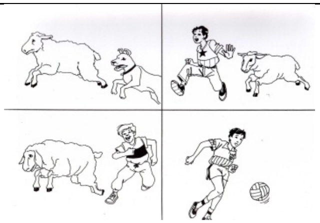
**Pronouns**	**OC -** L'éléphant les porte.<br>*The elephant carries them*	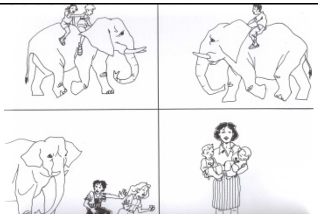
**Double negation**	**CO -** Ni le garçon ni le cheval ne courent.<br>*Neither the boy nor the horse run*	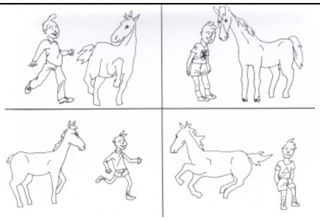
**Spatial relation**	**CO -** Le crayon est derrière la boîte.<br>*The pencil is behind the box*	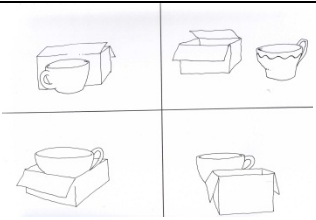
**Embedded relative clause with spatial relation**	**CO -** Le crayon qui est sur le livre est jaune.<br>*The pencil on the book is yellow*	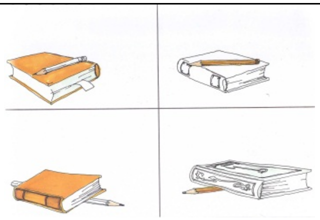
