# Supplementary material for: Beyond executive functions, creativity skills benefit academic outcomes: Insights from Montessori education
Source: PLoS One. 2019 Nov 21;14(11):e0225319. doi: 10.1371/journal.pone.0225319 (PMC6874078; doi:10.1371/journal.pone.0225319)
Supplement: S4 Fig — (PDF) [file pone.0225319.s007.pdf]

**Oral Sentence**	**Expected Answer**
“Jean a 4 cerises. Il en mange 1. Combien de cerises lui reste-t-il ?”<br>*Jean has 4 cherries. He eats one. How many cherries are left?*	4 – 1 = **3**
“Pierre a 12 billes. Il donne 5 billes à sa copine Anne. Combien de billes a Pierre maintenant ?”<br>*Pierre has 12 marbles. He gives 5 marbles to his girlfriend Anne. How many marbles has Peter now?*	12 – 5 = **7**
“Il y a 4 poissons dans le bocal. David ajoute des poissons. Maintenant, il y a 6 poissons dans le bocal. Combien David a-t-il ajouté de poissons ?”<br>*There are four fishes in the jar. David adds fishes. Now there are six fishes in the jar. How many fishes did David add?*	4 + x = 6<br>x = **2**
